# Effect of an anti-human Co-029/tspan8 mouse monoclonal antibody on tumor growth in a nude mouse model

**DOI:** 10.3389/fphys.2014.00364

**Published:** 2014-09-19

**Authors:** Naouel Ailane, Céline Greco, Yingying Zhu, Monica Sala-Valdés, Martine Billard, Ibrahim Casal, Olivia Bawa, Paule Opolon, Eric Rubinstein, Claude Boucheix

**Affiliations:** ^1^Inserm, UMR-S1004Villejuif, France; ^2^Université Paris-Sud 11Villejuif, France; ^3^Gustave Roussy, Laboratoire de Pathologie ExpérimentaleVillejuif, France

**Keywords:** tetraspanins, Co-029/tspan8, monoclonal antibodies, colorectal cancer, nude mice, therapy

## Abstract

New therapeutic agents are needed in digestive tract tumors. Co-029/tspan8 is a tetraspanin frequently expressed on human colorectal tumors, In this work, we report the effects of the monoclonal antibody Ts29.2, targeting Co-029/tspan8, on colorectal tumor cells *in vitro* and after implantation in nude mice. HT29, Isreco1 and SW480 colorectal tumor cell lines were used for this study. HT29 has a strong endogenous expression of Co-029/tspan8, whereas Isreco1 cells don't express Co-029/tspan8 and SW480 has only a weak expression. Isreco1 and SW480 were transduced to express Co-029/tspan8 at the same level as HT29. In order to check the specificity of the effect of monoclonal antibody Ts29.2, low Co-029/tspan8 expressing SW480 cells were injected simultaneously with transduced cells in the back, on the left and right sides of the mice. With an early treatment, Ts29.2 mAb inhibited growth of tumors expressing Co-029/tspan8 up to 70%, whereas a delayed treatment was less efficient. No effect of the antibody on cell proliferation or apoptosis induction was detected *in vitro*. No increase of activated caspase 3 labeling was observed *in vivo* and areas occupied by vessels were not significantly different between treated mice and controls. This suggests that the action of Ts29.2 is linked neither to cellular toxicity nor to the inhibition of the previously reported angiogenic properties of Co-029/tspan8. An inhibition of cell proliferation *in vivo* is demonstrated by a reduction of the mitotic index in HT29 tumors of Ts29.2 treated mice. The discrepancy between *in vitro* and *in vivo* data on cell proliferation suggests that the binding of Ts29.2 to tumor cells may modify their response to signals issued from the microenvironment. Given the restricted pattern of tissue expression of the tetraspanin Co-029/tspan8, these preliminary results put forth for consideration the antibody targeting of this tetraspanin in further investigations for therapeutic applications.

## Introduction

Colorectal cancer is one of the most frequent tumors worldwide and one of the leading causes of cancer-related deaths. Colorectal cancers account for 9% of all cancer related deaths in the USA. The 5-year relative survival rates of US patients is 64%. At early localized stages (39% of the patients at diagnosis), the 5-year survival rate is above 90% but if adjacent organs or lymph nodes are involved, it drops to 69% and to 12% if the disease is metastatic (American Cancer Society, 2012). Adjuvant therapy reduces the risk of relapse but it is only partially efficient and not without adverse effects. For metastatic tumors, cure expectation remains low even if it has greatly improved in case of resectable liver metastasis with the combination of more effective medical treatment combined with surgery (Bilchik et al., 2008).

Monoclonal antibodies have come of age as therapeutics in numerous diseases including cancer. Since the elimination of foreign antibodies by the immune system has been largely solved through humanization of murine antibodies, other problems remain to be overcome in diverse situations in order to get the most efficient and tolerated therapeutic drugs. Only a limited number of mAb are presently approved for therapy of solid tumors due to several factors, a major barrier being diffusion into the tumor. The antigenic target is also a matter of concern since the expression of this antigen may be harmful for the patient if it is targeted in normal tissues. Therefore, it is preferable that the antigen is either not expressed in normal tissues or in few tissues and at a low level as compared to the tumor. It is also of value if the expression of the antigen correlates positively with the prognosis.

Monoclonal antibodies are already widely used for treatment of colorectal cancer. Cetuximab, that targets EGFR, is a function blocking antibody and it's *in vivo* effect is probably linked to this property. In association with chemotherapy it induces a prolonged progression free survival whereas no effect on overall survival has been reported. The efficiency of Cetuximab is hampered by activating mutations of KRAS and BRAF thus limiting its use (Dahabreh et al., 2011; Lin et al., 2011). Bevacizumab that targets VGFR, is also efficient in colorectal tumors treatment. However, high blood pressure, diarrhea, mouth sores and delayed wound healing (Hompes and Ruers, 2011) are some of Bevacizumab's side effects while those of cetuximab include itching, acne-like skin rash and low blood electrolyte levels.

New antibodies targeting more specifically colorectal tumors antigens would therefore be of great help. Tetraspanin Co-029/tspan8 (Zoller, 2009) could be an appropriate target for mAb therapy in digestive tumors. The expression of this molecule is restricted to a small number of tissues such as digestive epithelial cells especially in colon and stomach and slightly on biliary epithelial cells. Its expression has been also reported in tumors and apart from esophagus, stomach and colorectal cancers, it can be observed in liver, prostate, ovarian and cervical cancer (Uhlen et al., 2010). In addition we have shown earlier that the expression of Co-029/tspan8 is associated with a poor prognosis in colorectal cancer (Greco et al., 2010). Similar observations have been made for esophageal (Zhou et al., 2008) and hepatocellular carcinoma (Kanetaka et al., 2001).

In the present study, we have used a new Co-029/tspan8 mAb produced in our laboratory to demonstrate an *in vivo* effect against human tumors engrafted in nude mice and have compared our observations with previous reports related to the biology of this tetraspanin.

## Materials and methods

### Cells and cell culture

The cell lines Isrecol, was initially derived from a primary human colon cancer (Duke's C, class III) surgical specimen (Isreco1) together with cell lines from its corresponding liver and peritoneal metastases, Isreco2 and Isreco3 (Cajot et al., 1997). The colorectal carcinoma cell line SW480 and HT29 were purchased from ATCC. The cell lines, were cultured in Dulbecco's modified Eagle's medium (DMEM) supplemented with 10% FCS, glutamax and antibiotics (all from Invitrogen). Isreco1 cells harbor a G12D homozygous mutation of KRAS whereas SW480 and HT29 cells were checked for respectively the KRAS homozygous G12V mutation and the BRAF V600E mutation.

### Lentiviral vectors

The human Co-029/tspan8 cDNA coding sequence was inserted in the TRIPΔ3-EF1α vectors. Vector particles were produced by cotransfection of 293T cells by the TRIPΔ3-EF1α-Co029 plasmids together with encapsidation and envelope (vesicular stomatitis virus) expression plasmids (Greco et al., 2010). Isreco1 cells and SW480 cells were transduced twice with concentrated lentiviral particles.

### Antibodies

The Co-029/tspan8 mAb TS29.2 (IgG2b), not reported before, was issued from the same fusion as Ts29 (an IgG1 that is now called Ts29.1) (Greco et al., 2010). Briefly, BALB/c mice were injected intraperitoneally twice with a mixture of 10^7^ Isreco3 and Lovo cells and a final boost was performed 3 weeks later with CD9-containing complexes collected by immunoprecipitation from a Brij97 lysate of 10^9^ Isreco3 cells that express strongly Co-029/tspan8 (Le Naour et al., 2006). Spleen cells were fused with P3X63AG8 mouse myeloma cells (5 × 10^7^ and 3 × 10^7^ cells respectively) according to standard techniques and distributed into 96-well tissue culture plates. After 2 weeks hybridoma culture supernatants were harvested and tested for Isreco1 and Is1-Co029 staining by indirect immunofluorescence. Positive supernatants were then further characterized by immunoprecipitation. The mAb Ts29.2 was purified by MEP Hypercel mixed-mode sorbent (Pall France) from ascitic fluids. Purity was checked by gel electrophoresis and Coomassie blue staining. Using infrared scanning of the gel with Odyssey Infrared Imaging System (LI-COR Biosciences), the purity was shown to be superior to 90%.

### Immunofluorescence

For flow cytometry analysis of cell surface molecules, cells were detached using a non-enzymatic solution (Invitrogen), washed and stained with 10 μg/ml of primary antibody. After washes in culture medium, cells were incubated with 10 μg/ml FITC-labeled secondary antibody (Beckman Coulter), washed again three times and fixed with 1% formaldehyde in PBS. All incubations were performed for 30 min at 4°C. Analysis of cell-surface staining was performed with an Accuri C6 flow cytometer (Becton-Dickinson, San Jose, CA, USA).

### Immunoprecipitation and western blot

Cells were lysed directly in the tissue culture flask (1 ml for a 75-cm2 flask) in lysis buffer (10 mM Tris (pH 7.4), 150 mM NaCl, 0.02% NaN3, 1 mM phenylmethylsulfonyl fluoride, 0.5 mg/ml leupeptin, 1 mg/ml pepstatin A and 10 kallikrein-inactivating units/ml aprotinin) containing 1% Triton X-100 (Roche Molecular Biochemicals, Meylan, France). After a 30-min incubation at 4°C, the insoluble material was removed by centrifugation at 10,000 g and the cell lysate was precleared overnight by addition of 0.005 volume of heat-inactivated goat serum and 0.025 volume of protein G-Sepharose beads (Amersham Pharmacia Biotech). Proteins were then immunoprecipitated by adding 2 μg/ml of antibodies and 10 μl of protein G-Sepharose beads (GE Healthcare) to 1 ml of the lysate. After a 2-h incubation at 4°C under constant agitation, the beads were washed five times in lysis buffer. The immunoprecipitates were separated by 5–15% SDS-polyacrylamide gel electrophoresis under non-reducing conditions and transferred to a PVDF membrane (Amersham). Western blotting on immunoprecipitates was performed using biotinylated Ts29.2 and a Alexa Fluor 680-labeled streptavidin (Invitrogen) which was revealed with the Odyssey equipment.

### Effect of Ts29.2 mAb on cell growth and apoptosis *in vitro*

Cells in complete FCS medium were distributed into 6 well plates at 20,000 cells/well. Cells were divided into three groups with 0, 10, and 50 μg/ml of Ts29.2. Cell counts were performed in duplicated wells at 24, 48, and 72 h. At 72 h, apoptotic cells were also quantified by flow cytometry using propidium iodide (PI, Sigma). After overnight fixation in ethanol 70% at −20°C, PI at 50 μg/ml was added and apoptosis was measured by counting cells with sub-G1 DNA content. Statistical analysis were performed using GraphPad Prism software. Repeated measure ANOVA was used for comparison between the 3 groups and calculation of the *p*-value.

### *In vivo* experiments

Balb/c nude mice were injected with 5–10.10^6^ tumor cells subcutaneously in the back on day 0. For SW480, when indicated, each mouse was implanted with the Co-029 weakly expressing SW480 cell line on one side and with the corresponding transduced Co029 cell lines on the right side. Mice were divided between treatment and control groups, each group comprising 5 mice. The treated mice were given the Ts29.2 mAb as follows: a first injection of 2 mg of antibody was given intraperitoneally on day 0 and 1 mg was given intraperitoneally twice a week, generally for 4 weeks. Other schedules were tested, single early injection for SW480-Co029 tumors, or delayed treatment for HT29 tumors as reported in the Results Section. Control mice received the same volume of PBS intraperitoneally. The size of the tumor was determined with the formula (π/6 × length × width × thickness). U-Mann-Whitney test was performed for data analysis. Experiments were conducted according to the French veterinary guidelines and those formulated by the European Commission for experimental animal use (L358-86/609EEC) and were approved by Inserm (National Institute for health and medical research, France).

### Biolocalization

Tumors in nude BALB/c mice were induced by subcutaneous injection of 10^7^ tumor Isreco-Co029 or SW480-Co029 cells. After 10 days, 4 mice were injected intraperitoneally with 1 mg Ts29.2 mAb. Two mice were sacrificed by cervical dislocation at 24 and 48 h after injection of the mAb. Tumors were excised and included in tissue freezing medium (Tissue-Tek, Sakura Finetek Europe) and immediately frozen in liquid nitrogen vapors. Frozen sections were stained using anti-mouse Alexa 488 labeled goat antibodies (Molecular Probes, USA) to detect the presence of mAb in the tumors.

### Histopathology and immunohistochemistry for angiogenesis, apoptosis and proliferation analysis

Following fixation of the tumors in 4% paraformaldehyde, paraffin sections (4 μm thick) were prepared. For pathological examination, sections were stained with hematoxylin and eosin. Immunohistochemistry for anti-CD34 positive murine endothelial cells (HyCult biotechnology b.v., The Netherlands) was performed after xylene treatment and rehydration of paraffin sections. Heat-induced epitope retrieval was achieved with pH8 Tris-EDTA at 98°C for 30 min. Endogenous peroxidase activity was quenched by 3% H_2_O_2_ for 10 min. The sections were placed in coverplates (Shandon, U.S) and incubated with blocking serum Biogenex 1:20 (San Ramon, Ca) for 10 min. This step was followed by incubation with anti-mouse CD34 1:20 diluted in blocking serum 1:10 during 1 h. Slides were then incubated with a rabbit anti-rat 1:400 (Southern Biotech., Al., USA) diluted in blocking serum 1:10. The following step consisted in a Rabbit PowerVision kit (ImmunoVision Technologies, Ca) 20 min and DAB 10 min. Slides were counterstained with Mayer's hematoxilin and mounted (Pertex). Quantification of positive vessels was assessed with a method combining a dedicated slide scanner and a computer-assisted image analysis (Kim et al., 2003). The positive signal was expressed by the area fraction of stained endothelial cells within the tumor compared to the whole surface of the tumor, selecting only viable tissue. This procedure was achieved using Pix-Cyt, a software package designed by the Groupe Régional d'Etudes sur le Cancer, Centre François Baclesse, Caen. The same protocol was used for quantifying cells expressing activated caspase 3. An antihuman activated caspase 3 antibody was used at 1:100 (Cell Signaling, Ozyme, France). The mitotic index was calculated as the percentage of mitotic figures per total tumor cells in two separate fields for each HES stained slides of HT29 tumors. At least 1000 cells were counted for each tumor. U-Mann-Whitney test was performed for data analysis.

## Results

### Generation of Isreco1 and SW480 colon carcinoma cell lines expressing Co-029/tspan8

**Figure 1:**
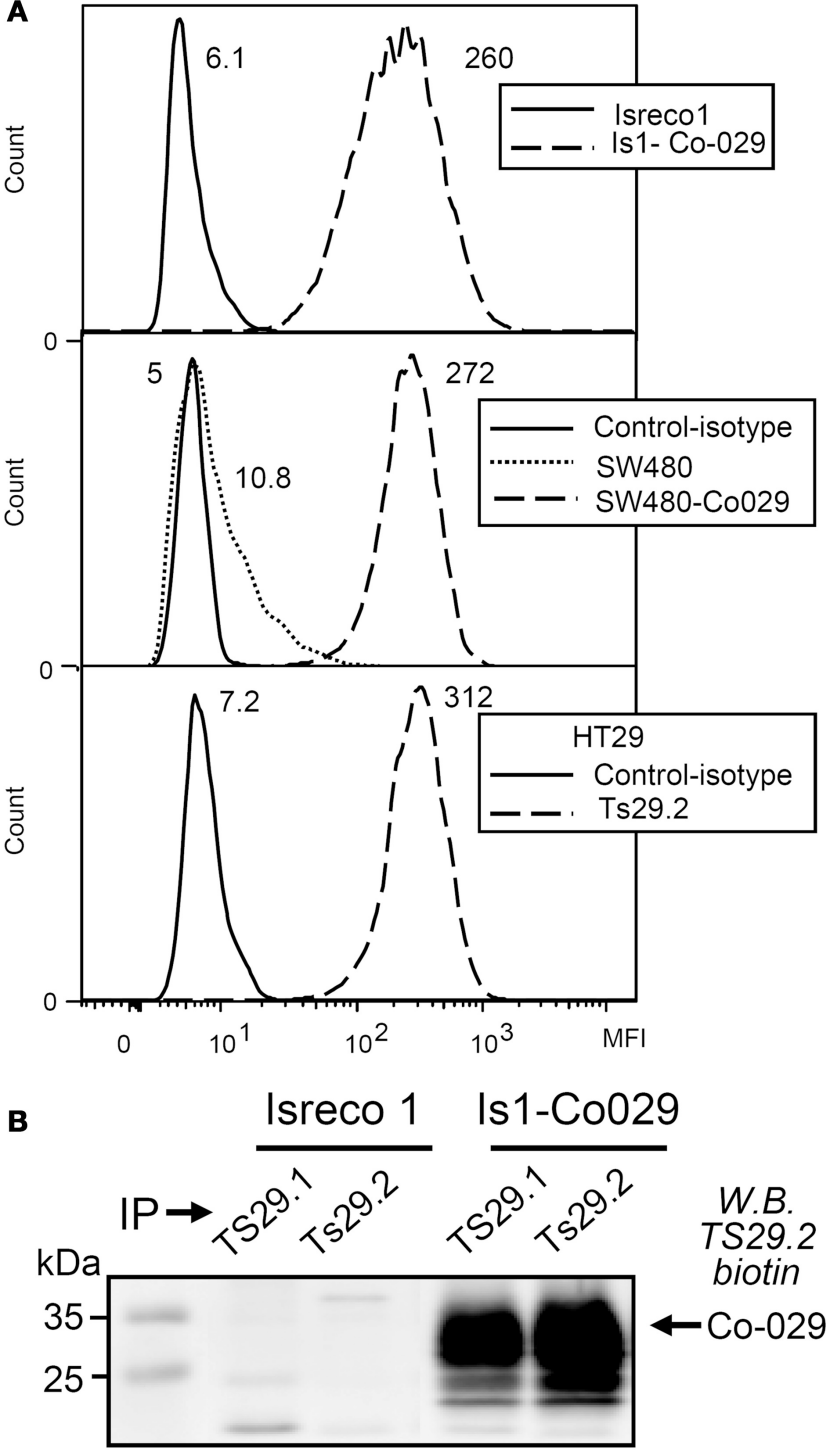
**Co-029/tspan8 recognition by mAb Ts29.2. (A)** Expression of Co-029/tspan8 by transduced cell lines Isreco1 and SW480 as compared to HT29 cell line. A similar level of expression was obtained after transduction compared to endogenous expression of HT29 cells. SW480 cells have a low endogenous expression of Co-029/tspan8. Negative isotypic controls of Isreco1 and Is1-Co029 are not shown because they are at the same level as Isreco1 labeled with Ts29.2 mAb. Mean Fluorescence Intensity values appear directly on the graphs **(B)** Western blot with biotinylated-Ts29.2 mAb of cell extracts immunoprecipitated by Ts29.1 (previously Ts29) or Ts29.2.

For *in vitro* and *in vivo* studies, the Co-029/tspan8 negative Isreco1 and SW480 cells were transduced with lentiviral vectors in order to obtain an expression of Co-029/tspan8 similar to HT29 cells and metastatic cell line Isreco2 (Greco et al., 2010) (Figure 1A). The specificity of Ts29.2 as compared to the previously used Ts29.1 mAb was further checked by immunoprecipitation and western blot (Figure 1B).

### Effect of Ts29.2 mAb on cell proliferation and apoptosis *in vitro*

**Figure 2:**
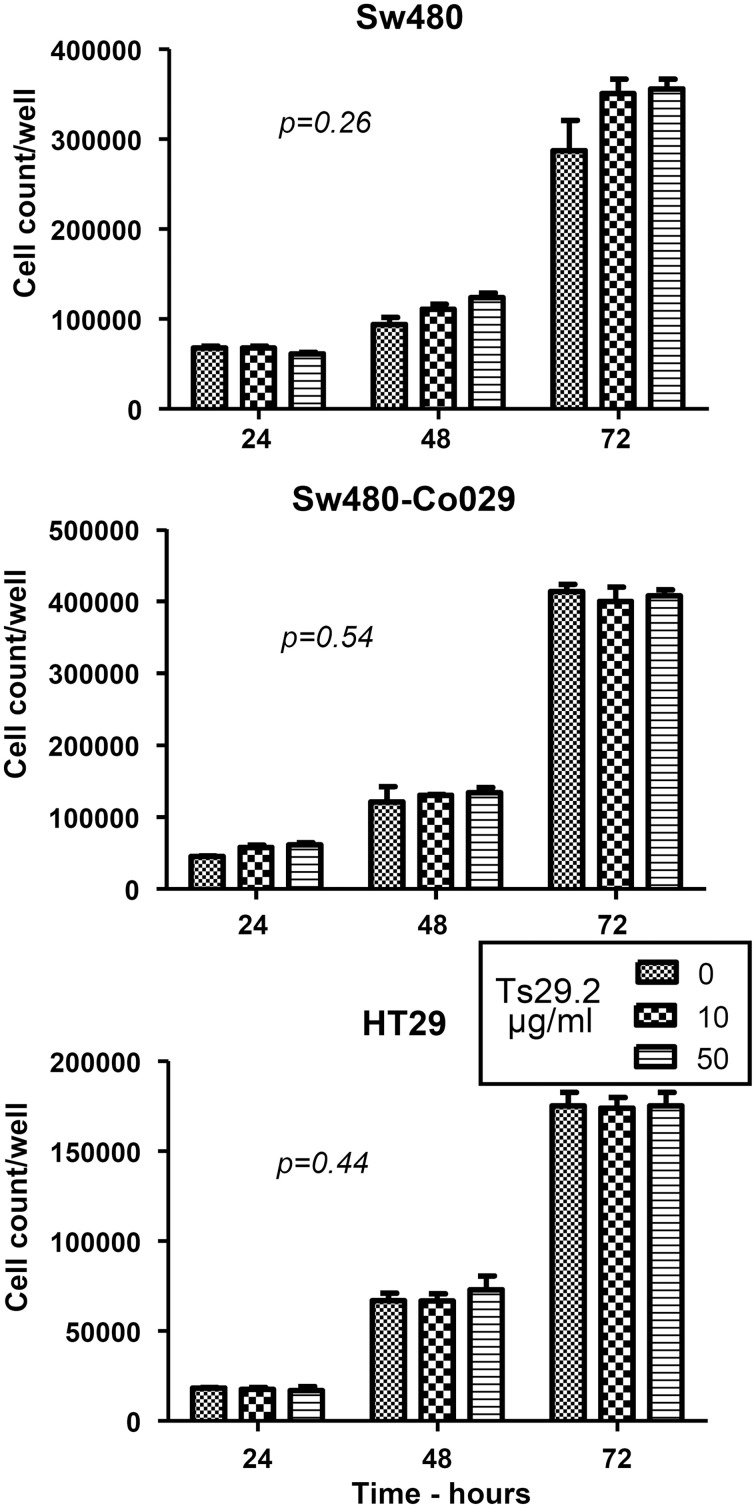
**Effect of Ts29.2 mAb on cell proliferation**. Ts29.2 mAb has no effect on cell proliferation at 10 or 50 μg/ml on cells expressing either a low or a high level of tetraspanin Co-029/tspan8.

SW480 and SW480-Co029 cells were seeded at 20000/well in 6 well culture plates. After 12 h to let cells adhere to the plates, Ts29.2 mAb was added at concentrations of 10 and 50 μg/ml. Cell counts were performed 24, 48, and 72 h later. No difference in proliferation was observed between SW480, SW480-Co029, and HT29 cells cultured in the presence or absence of Ts29.2 mAb (Figure 2). Results shown are representative of 2 experiments. An absence of effect on proliferation was also observed for Isreco1 and Is1-Co029 cells (data not shown). At 72 h, an apoptosis test was performed that showed a proportion of apoptotic cells below 5%, similar between control and antibody treated cells (data not shown).

### Biolocalization of Ts29.2 mab in xenografts

**Figure 3:**
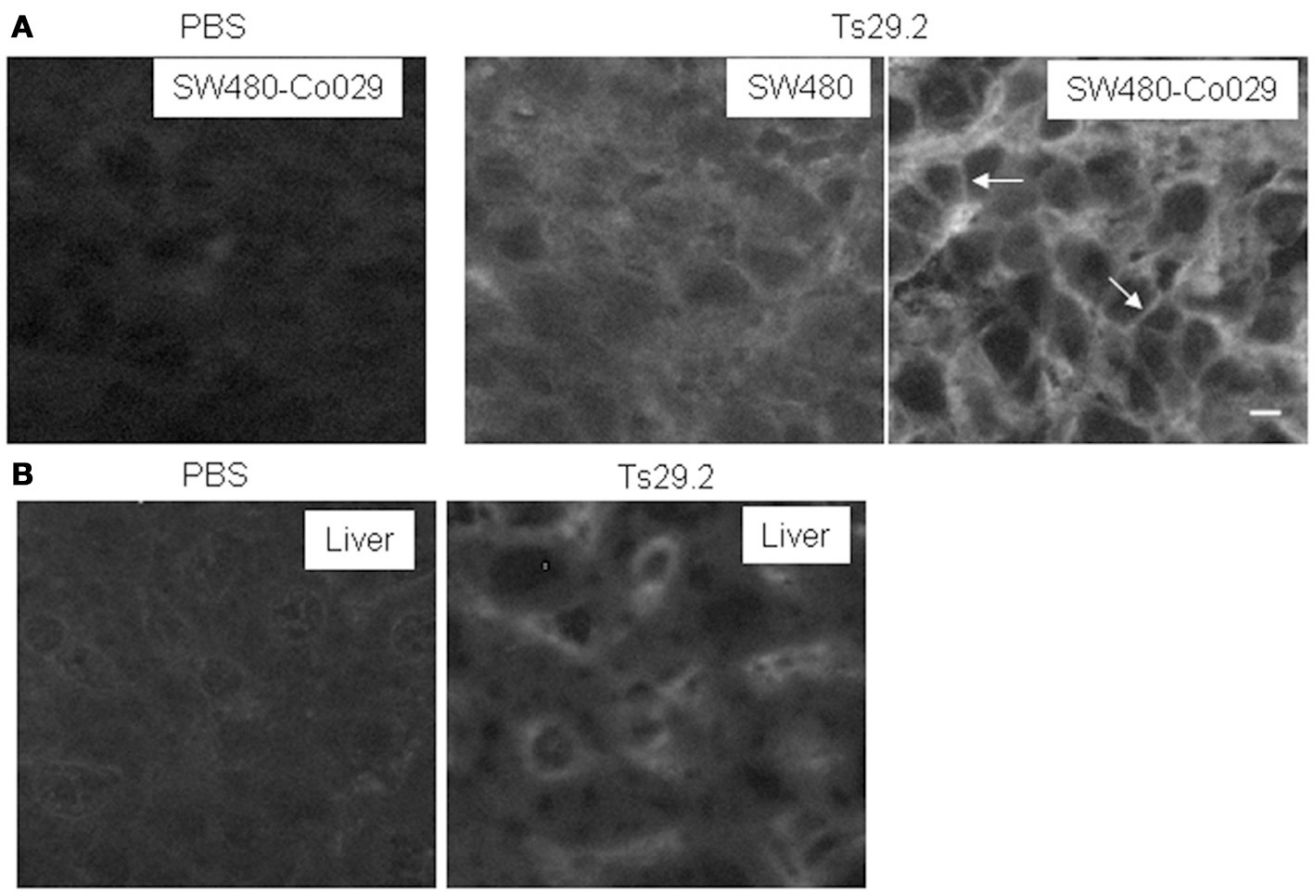
**Detection of the TS29.2 mAb *in vivo* 24 h after injection**. **(A)** SW480-Co029 tumor cells are strongly labeled. This labeling appears to be predominantly membranous (arrows). PBS is shown as negative control. In SW480 tumors, a weaker staining with a diffuse interstitial labeling is visible showing that the mAb penetrates inside the tumor. **(B)** Due to the presence of circulating Ts29.2 mAb sinusoids are labeled in the liver of injected nude mice. PBS is shown as negative control. Scale bar 10 μm.

Localization of the mAb into the tumor is a requirement for its use for therapeutic purpose. Therefore, we analyzed intratumoral distribution after i.p. injection of 1 mg Ts29.2 mAb in mice carrying previously untreated tumors. Frozen sections of tumors excised at 24 and 48 h after mAb injection were scored for mAb influx by immunofluorescent staining. A weak diffuse extracellular labeling was seen in SW480 tumors. An intense surface labeling was detectable only on Co-029/tspan8 expressing tumor cells (Figure 3A). The antibody was also detected in liver sinusoids (Figure 3B). Similar aspects were observed at 24 and 48 h.

### Early treatment of tumors with multiple injections of Ts29.2 mAb

**Figure 4:**
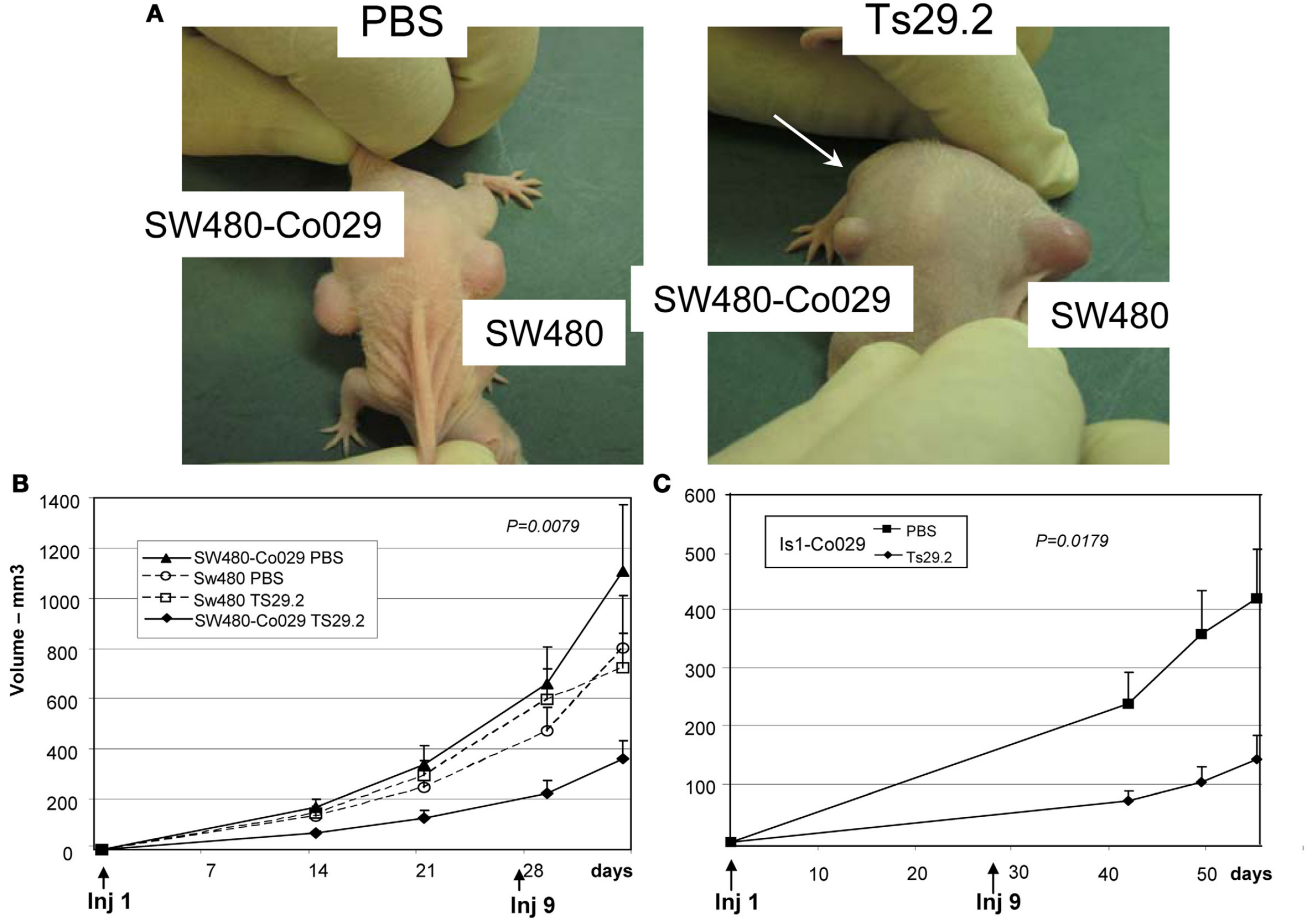
**Effect of Ts29.2 mAb treatment on tumor growth *in vivo*. (A)** A clear inhibition of SW480-Co029 growth is visible (arrow) in the mouse treated with Ts29.2mAb as compared to SW480 tumor (right) or tumors in PBS treated mice (left). **(B)** Growth curves of SW480-Co029 tumors in nude mice compared to tumors treated with PBS or SW480 cells expressing a low level of Co-029/tspan8. First and last injections of Ts29.2 mAb or PBS are indicated below. **(C)** Growth curves of Is1-Co029 tumors in nude mice treated with Ts29.2 mAb. First and last injections of Ts29.2mAb or PBS are indicated below.

Ten Balb/c nude mice were injected with 10^7^ tumor cells subcutaneously on the back, on the left side for SW480 cells and on the right side for SW480-Co029 cells and the treatment was immediately started. SW480 tumors grew rapidly and already at day 14, a clear inhibition of growth of SW480-Co029 tumors in the Ts29.2 treated group was observed as compared to the PBS control groups. Globally, the reduction of growth was above 70%. Growth of SW480 tumors was similar in PBS and Ts29.2 treated groups and the mean size was slightly inferior to PBS treated SW480-Co029 tumors (Figures 4A,B).

Nude mice were also injected with Is1-Co029 tumor cells and treated according to the same protocol. Tumor growth was much slower than for SW480 tumors and measurements started at day 42. However, a similar inhibition linked to antibody treatment was observed (Figure 4C). The expression of Co-029/tspan8 did not change significantly the tumor growth pattern (data not shown).

**Figure 5:**
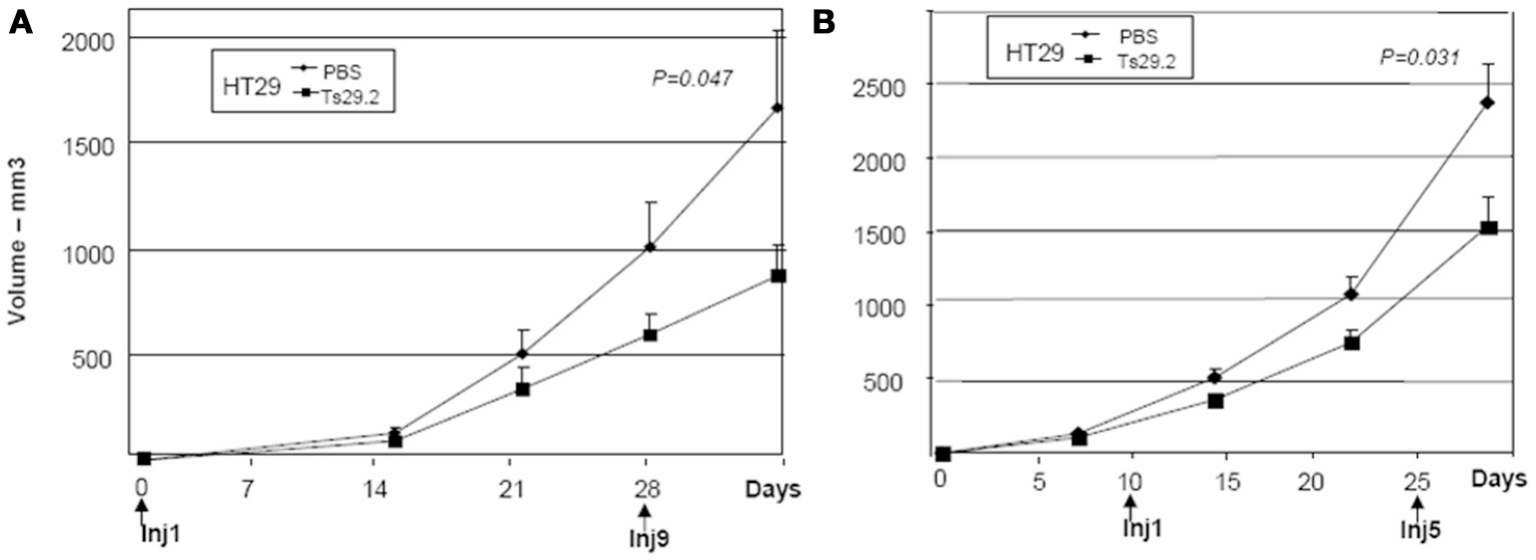
**Initial and delayed treatments of HT29 tumors in nude mice. (A)** Treatment started at day 0. An inhibition of 50% was observed after 4 weeks of Ts29.2 mAb injections. First and last injections of Ts29.2mAb or PBS are indicated below. **(B)** Same experiment but injections of Ts29.2 mAb started at day 10 after tumor implantation. A lower inhibition (40%) was observed. First and last injections of Ts29.2 mAb or PBS are indicated below.

The endogeneously Co-029/tspan8 expressing HT29 cells grew very fast in nude mice. Two groups of five mice were injected with 5.10^6^ cells subcutaneously and the same treatment protocol was applied. An inhibition of 50% of tumor growth was observed (Figure 5A).

Interestingly, the expression of Co-029/tspan8 did not change the *in vivo* growth of PBS treated SW480 tumors (Figure 4B) or Isreco1 tumors (data not shown).

Histological examination showed encapsulated tumors with large areas of necrosis in all tumors (Figure S1) whose general aspect did not differ between Ts29.2 mAb and PBS treated mice (Figure S2). Moreover, no significant infiltration by inflammatory cells could be noticed in Ts29.2 treated mice as compared to PBS controls, whether the cells expressed Co-029/tspan8 or not. This picture differed from what was observed in tumors implanted in nude mice in which a strong ADCC was observed following injection of a mAb of IgG2a subclass, a subclass with strong ADCC activity. These tumors were essentially necrotic with the presence of large foamy macrophages (Adams et al., 1984).

### Treatment of tumors with a single injection of mAb

In order to determine if the early injection of 2 mg Ts29.2 mAb could inhibit tumor implantation, two groups of five mice were treated with a single dose of Ts29.2 mAb at the time of SW480-Co-029/tspan8 tumor implantation. No difference was noticed between tumors treated with Ts29.2 or PBS indicating that a sustained treatment is required for an observable effect and that an effect on tumor implantation cannot be invoked for inhibition of tumor growth (data not shown).

### Delayed treatment of tumors

The effect of delayed treatment with Ts29.2 mAb on HT29 tumor growth was also evaluated. For that purpose, 25 nude mice were injected with 5.10^6^ cells on day 0. On day 10, 10 mice with tumors of similar size were selected and separated in 2 groups. One group was injected with 2 mg Ts29.2 followed by injections of 1 mg twice a week for 2 weeks while the other group of mice received control PBS injections. An inhibition of growth of 40%, slightly lower than with the early starting treatment (Figure 5A), was observed (Figure 5B).

### Angiogenesis, apoptosis, and proliferation *in vivo* (Figure 6)

**Figure 6:**
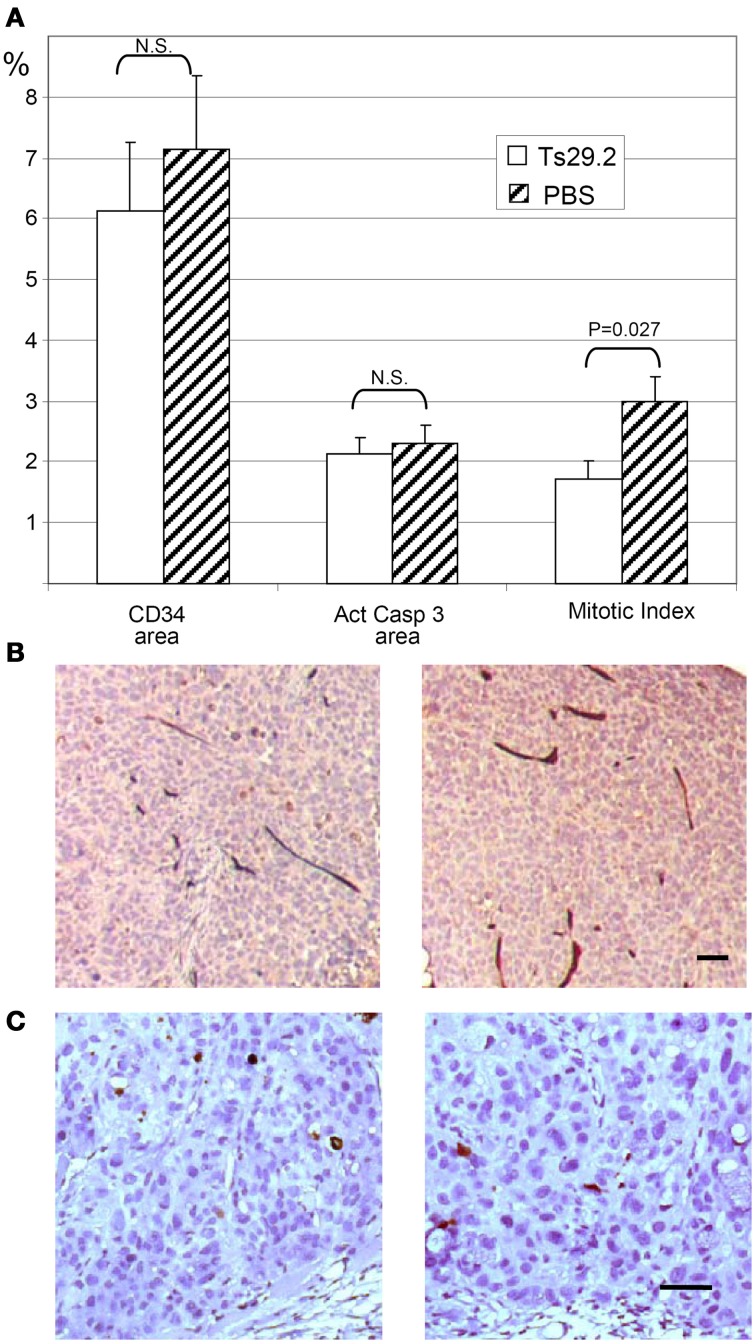
***In vivo* effect of HT29 tumors treatment with Ts29.2 antibodies**. **(A)** Quantification of angiogenesis (CD34 vascular labeling), apoptosis (activated caspase 3 labeling) and proliferation (mitotic index). **(B)** DAB stained vessels are clearly seen within neighboring tumor areas. **(C)** The DAB stained cells are easily distinguished from neighboring tumor areas. No difference is seen between the mAb Ts29.2 treated tumor (left) and the PBS treated tumor (right) in **(B,C)**. Scale bar 50 μm.

In order to check for an effect of Ts29.2 mAb on tumor angiogenesis as was reported for an anti-rat Co-029/tspan8 mAb (Claas et al., 1998; Gesierich et al., 2006), we analyzed vascular density by CD34 labeling and image analysis. No significant differences were observed between Ts29.2 HT29 *in vivo* treated tumors and controls (Figures 6A,B). Similarly, *in situ* quantification of apoptosis by activated caspase 3 labeling did not show a difference between PBS and Ts29.2 treated HT29 (Figures 6A,C) or SW480-Co029 tumors (not shown). However, an effect of Ts29.2 on cell proliferation was detected since the mitotic index was 1.71% ± 0.31% in the tumors cells of Ts29.2 treated mice whereas it was 3% ± 0.4% for PBS treated mice (*p* = 0.027) (Figure 6A).

## Discussion

This work shows that an IgG2b mouse mAb toward human tetraspanin Co-029/tspan8 slows down significantly growth of colorectal tumors *in vivo* in a xenogeneic nude mice model.

Tetraspanins are membrane proteins forming a 33 family members whose function has not been elucidated apart from their ability to form large multimolecular membrane complexes, the “tetraspanin web” (or tetraspanin enriched microdomains) and for their control over traffic or function of some associated molecules (Hemler, 2003; Charrin et al., 2009). A relation between tumor prognosis and the level of expression of several tetraspanins has been widely reported, either in patients or experimentally, in numerous tumors (Boucheix and Rubinstein, 2001; Charrin et al., 2009; Zoller, 2009; Romanska and Berditchevski, 2011; Hemler, 2013) while opposite effects have been described depending on the tetraspanin studied. As a matter of fact, the way the expression of a tetraspanin may affect the course of a tumor remains widely unknown and may differ from one tetraspanin to another. It is hypothesized that tetraspanin effects are mediated by molecular partners within the “tetraspanin web.” Among tetraspanins that were reported as modulators of tumor progression, CD82 is considered as a metastasis suppressor gene (Dong et al., 1995) and associated with favorable prognosis through an inhibitory effect on cell motility and invasiveness. High CD9 expression has also mostly been associated with a favorable prognosis for patients and reduced metastasis in animal models (Ikeyama et al., 1993; Boucheix et al., 2001; Zoller, 2009). Also, intratracheal administration of adenovirus encoding either MRP-1/CD9 or KAI1/CD82 into lung tumors-bearing mice reduced metastasis to the mediastinal lymph node (Takeda et al., 2007). There is even a synergy in simultaneous reduction of CD9 and CD82 that leads to increased metastatic potential in breast cancer (Huang et al., 1998). On the other hand high levels of tetraspanins CD151 and Co-029/tspan8 are associated with poor prognosis (Zoller, 2009; Greco et al., 2010). Possible mechanisms for a role of CD151 overexpression in tumor progression may come from an enhancement of Rac and cdc42 activation (Shigeta et al., 2003) and an effect of integrin-mediated tumor cell motility via FAK activation (Kohno et al., 2002). Only few experiments with tetraspanin knockout mice were reported but confort previous experimental and clinical data. In the TRAMP (Transgenic adenocarcinoma of mouse prostate) model, genetic ablation of the tetraspanins *Cd9* and *Cd151* did not affect prostate tumor initiation but *Cd151* ablation reduces spontaneous metastatic spread whereas ablation of the tetraspanin *Cd9* increases spontaneous metastases, in an organ specific manner in both cases (Copeland et al., 2013a,b).

Co-029/tspan8 protumoral properties have been clearly shown by Zoller and coworkers (Gesierich et al., 2006) in a rat model of pancreatic tumor. *In vitro* rat Co-029/tspan8 has been shown to promote motility through its association with α6β 4, to increase apoptosis resistance and in an *in vivo* hepatoma model to promote liver metastasis (reviewed in Zoller, 2009). Conversely, in the absence of integrin α6β 4, the rat tspan8 D6.1A, appears to be a strong inducer of angiogenesis. In this model, the angiogenic effect appears to be mediated by tumor cells exosomes that recruit tspan8 together with other tetraspanins like CD9 or CD151 and associated molecules (Nazarenko et al., 2010). In our model of colorectal Co-029/tspan8 transduced tumor cell lines, we were not able to demonstrate a direct effect of Co-029/tspan8 on cell motility on collagen I (Greco et al., 2010). However, such an effect became apparent *in vitro* after silencing of E-cadherin, p120ctn or RhoA in Isreco cell lines. By crosslinking, we have shown that Co-029/tspan8 may associate with E-cadherin thus providing a molecular basis for this observation. In nude mice, the growth of Isreco1 or SW480 tumors and their corresponding Co-029/tspan8 transduced cell lines was either identical or not significantly different. In this context, analysis of vascular density didn't appear to be reduced in Ts29.2 mAb treated tumors compared to PBS treated tumors.

Concerning the *in vivo* effect of the mouse anti Co-029/tspan8 IgG2b Ts29.2, the inhibition of tumor growth occurs without toxic effect of the mAb on cells *in vitro* and in the absence at histological level of inflammatory cells infiltration in the tumor. Moreover, no significant increase of activated caspase 3 labeling was observed in HT29 treated tumors. This is in agreement with early observations that mouse IgG2b, as opposed to IgG2a, are considered as being poor mediators of ADCC in nude mice (Herlyn and Koprowski, 1982; Anasetti et al., 1987). However, we can't completely exclude a limited recruitment of effector cells that could be demonstrated by cellular dissociation of the tumors. Finally, since we could not demonstrate an effect on angiogenesis in Ts29.2 mAb treated mice another mechanism should be operating. These negative results contrast with another model of gastric tumor implantation in SCID mice treated with the mAb ALB6 (IgG1) directed against tetraspanin CD9 that resulted in inhibition of tumor growth associated with increased apoptosis and reduced angiogenesis (Nakamoto et al., 2009). On the other hand, the *in vivo* inhibition of proliferation assessed by the mitotic index in contrast to the absence of *in vitro* effect places an indirect functional effect of the mAb Ts29.2 on tumor growth among the likely hypotheses. Considering the role of tetraspanins in the assembly of cell surface multimolecular complexes containing various molecules such as integrins or growth factor receptors, signalization through these molecules appears as a possible target. An effect of tetraspanins on growth factor receptor signalization was already reported *in vitro* for CD82 that negatively regulates EGFR (Odintsova et al., 2000) but remains to be demonstrated for Co-029/tspan8. In addition, the MAP kinase pathway is already engaged due to the KRAS or BRAF mutations in the tumor cell lines used in this work. Therefore, as far as EGFR could be involved, an effect through another pathway as the PI3K-Akt pathway should be considered. Alternatively different growth factor receptors of the Erb family or other growth factor receptors like c-Met (Takahashi et al., 2007) could be turned on or regulated. The tetraspanin CD151 has also been tested as target of anti-tumor antibodies in animal models (reviewed in Haeuw et al., 2011). An inhibition of tumor growth has been observed *in vivo* with selected antibodies but the mechanism remains to be determined. Of interest is the inhibition by CD151 antibodies of tumor cell intravasation and metastasis formation in the chick embryo (Zijlstra et al., 2008).

To our knowledge, among tetraspanins, only CD37 has been targeted in human malignancies (Press et al., 1989; Sala-Valdes et al., 2012). An ^131^I conjugated mouse CD37 mAb was shown to induce complete remission in 6/6 patients with B cell lymphoma (Kaminski et al., 1993). Several humanized CD37 genetically engineered antibodies are currently under investigation for clinical applications (Heider et al., 2011; Krause et al., 2012). Their effects are mediated by apoptosis induction, ADCC (Heider et al., 2011) or direct cell death (Krause et al., 2012; Lapalombella et al., 2012).

Even in the absence of a clear mechanism for growth inhibition by Ts29.2 mAb, we propose that the tetraspanin Co-029/tspan8 can be an appropriate target for treatment of some epithelial tumors, especially colorectal tumors. The reasons are that it is a membrane protein for which antibodies are available, that its tissue distribution is restricted and that its increased expression is linked to a bad prognosis. Among tetraspanins and apart from CD37 in B cell malignancies, Co-029/tspan8 is one of the best candidates since other tetraspanins like CD9, CD82, or CD151 have a much wider tissue distribution. Toxicity of the biological drug may result from the destruction of normal cells and too much expression on normal tissues may trap the mAb and prevents its binding to tumor tissues.

Finally, the efficiency of the mAb could be considerably improved by replacing the Fc fragment with already available sequences and the immunization risk could be alleviated by humanization of the molecule. For all these reasons, targeting the tetraspanin Co-029/tspan8 is worth further developments in order to determine if it would be an efficient addition to currently available treatments of digestive tumors.

## Author contributions

All authors agree to be accountable for all aspects of the work, have approved the final version and provided substantial contribution to the work. Naouel Ailane performed most of the experiments with the help of Céline Greco, Yingying Zhu, and Monica Sala-Valdés and contributed to the writing. Ibrahim Casal supervised *in vivo* experiments. Pathological studies were performed by Paule Opolon and Olivia Bawa. The Ts29.2 antibody was produced and characterized by Martine Billard, Céline Greco, Eric Rubinstein and Claude Boucheix. Claude Boucheix designed the study and wrote the article that was revised by Eric Rubinstein and Paule Opolon.

### Conflict of interest statement

Patent—EP 10305818.6– Methods for cancer management targeting Co-029. The authors declare that the research was conducted in the absence of any commercial or financial relationships that could be construed as a potential conflict of interest.

## Abbreviations

mAb: monoclonal antibody
ADCC: antibody dependent cell cytotoxicity.
