# Supplementary material for: Effect of an anti-human Co-029/tspan8 mouse monoclonal antibody on tumor growth in a nude mouse model
Source: Front Physiol. 2014 Sep 19;5:364. doi: 10.3389/fphys.2014.00364 (PMC4168815; doi:10.3389/fphys.2014.00364)
Supplement: Supplementary file 1 [file Presentation1.PDF]

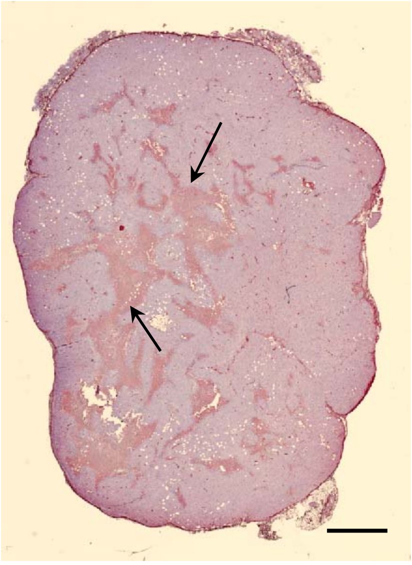

Supplementary Fig. S1: **Macroscopic aspect of a hematoxylin stained SW480 tumor subcunaeous nodule at low magnification.** Dense tumor proliferation was observed with large areas of tumor necrosis (arrows). Scale bar 1 mm.

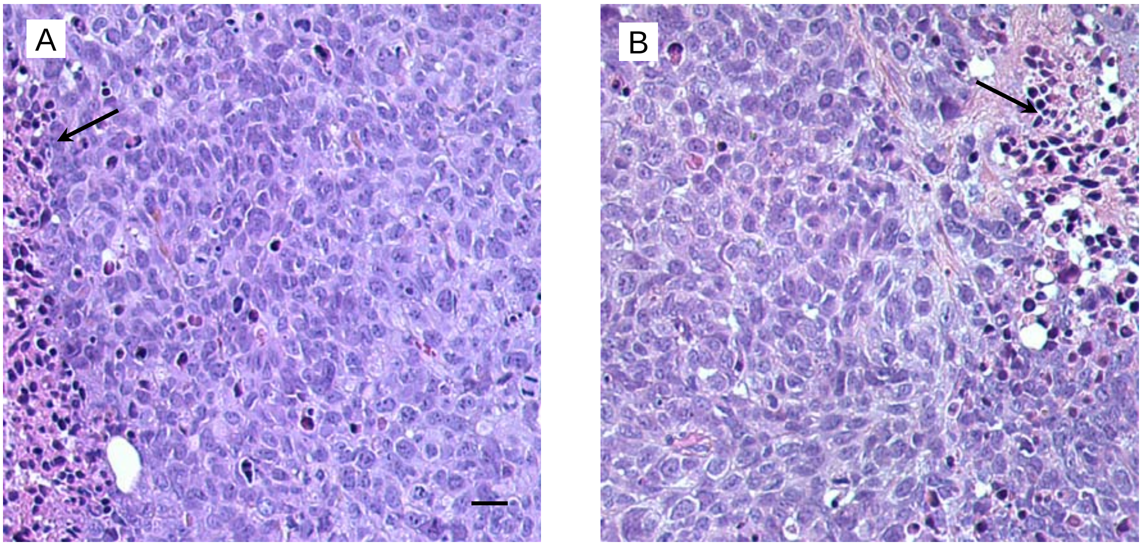

Supplementary Fig. S2: **Histological aspect of a SW480-Co029 treated tumors (HES staining).** Packed tumor cells with neighbouring areas of tumor necrosis (arrows) are visible. No difference is seen between the mAb Ts29.2 treated tumor (**A**) and the PBS treated tumor (**B**). Scale bar 20 µm.
